# Asiatic Cholera

**Published:** 1846-12

**Authors:** 


*Asiatic Cholera.*—This disease, which made its appearance at Aden early in May, has, in consequence of the changing of the monsoon, nearly vanished, isolated cases occurring only at intervals. During the five days it raged, upwards of four hundred persons were carried off, the deaths being four out of five attacked; the cholera is, however, rapidly advancing along the territory of Yemen, and fears may be entertained of its appearance on the shores of the Mediterranean. The disease is making dreadful havoc in India.—*Med. Times.—Ibid.*

